# Pathological Changes Following Neoadjuvant Endocrine Therapy (NAET): A Multicentre Study of 391 Breast Cancers

**DOI:** 10.3390/ijms25137381

**Published:** 2024-07-05

**Authors:** Islam M. Miligy, Nahla Badr, Andrea Stevens, David Spooner, Rachna Awasthi, Yasmeen Mir, Anuj Khurana, Vijay Sharma, Usha Chandaran, Emad A. Rakha, Yasmine Maurice, Daniel Kearns, Rami Oweis, Amal Asar, Alastair Ironside, Abeer M. Shaaban

**Affiliations:** 1 Cellular Pathology, Queen Elizabeth Hospital, Birmingham B15 2GW, UK; islam.miligy@yahoo.com (I.M.M.); rachna.awasthi@uhb.nhs.uk (R.A.); daniel.kearns@uhb.nhs.uk (D.K.); 2 Histopathology Department, Faculty of Medicine, Menoufia University, Shebin El-Kom 11352, Egypt; nahla.badr@med.menofia.edu.eg; 3 Oncology Department, Queen Elizabeth Hospital, Birmingham B15 2GW, UK; andrea.stevens@uhb.nhs.uk (A.S.); david.spooner@nhs.net (D.S.); 4 Pathology, Liverpool University Hospitals NHS Foundation Trust, Liverpool L7 8XP, UK; yasmeen.mir@liverpoolft.nhs.uk (Y.M.); anuj.khurana@liverpoolft.nhs.uk (A.K.); vijay.sharma@liverpoolft.nhs.uk (V.S.); 5 Histopathology Department, Salford Royal Hospital, Salford M6 8HD, UK; usha.chandran@nca.nhs.uk; 6 Histopathology Department, Nottingham City Hospital, Nottingham NG5 1PB, UK; emad.rakha@nottingham.ac.uk; 7 Histopathology Department, Heartlands General Hospital, Birmingham B9 5SS, UK; yasmine.maurice@swft.nhs.uk; 8 Histopathology Department, Rotherham Foundation Trust, Rotherham S60 2UD, UK; rami.oweis@nhs.net (R.O.); amal.asar@nhs.net (A.A.); 9 Department of Pathology, NHS Lothian, Edinburgh EH41 3PF, UK; alastair.ironside@nhslothian.scot.nhs.uk; 10 Institute of Cancer and Genomic Sciences, University of Birmingham, Birmingham B15 2SY, UK

**Keywords:** neoadjuvant endocrine therapy (NAET), breast cancer, pathological response, oestrogen receptor, progesterone receptor, HER2

## Abstract

Oestrogen receptor (ER)-positive breast cancer (BC) is generally well responsive to endocrine therapy. Neoadjuvant endocrine therapy (NAET) is increasingly being used for downstaging ER-positive tumours. This study aims to analyse the effect of NAET on a well-characterised cohort of ER-positive BC with particular emphasis on receptor expression. This is a retrospective United Kingdom (UK) multicentre study of 391 patients who received NAET between October 2012 and October 2020. Detailed analyses of the paired pre- and post-NAET morphological changes and hormone receptor (HR) and human epidermal growth factor receptor 2 (HER2) expression were performed. The median duration of NAET was 86 days, with median survival and overall survival rates of 380 days and 93.4%, respectively. A total of 90.3% of cases achieved a pathological partial response, with a significantly higher rate of response in the HER2-low cancers. Following NAET, BC displayed some pathological changes involving the tumour stroma including central scarring and an increase in tumour infiltrating lymphocytes (TILs) and tumour cell morphology. Significant changes associated with the duration of NAET were observed in tumour grade (30.6% of cases), with downgrading identified in 19.3% of tumours (*p* < 0.001). The conversion of ER status from positive to low or negative was insignificant. The conversion of progesterone receptor (PR) and HER2 status to negative status was observed in 31.3% and 38.1% of cases, respectively (*p* < 0.001). HER2-low breast cancer decreased from 63% to 37% following NAET in the paired samples. Significant morphological and biomarker changes involving PR and HER2 expression occurred following NAET. The findings support biomarker testing on pre-treatment core biopsies and post-treatment residual carcinoma.

## 1. Introduction

Oestrogen receptor (ER)-positive, human epidermal growth factor receptor 2 (HER2)-negative breast cancers (BCs) are less likely to respond to neoadjuvant chemotherapy (NACT) [1,2]. Endocrine therapy remains the mainstay for ER-positive BC patients and a valid alternate option for this group of patients in the neoadjuvant setting [2]. 

Previous studies have demonstrated that neoadjuvant endocrine therapy (NAET) is an alternative to NACT for ER-positive, HER2-negative tumours either for downstaging tumours or for delaying surgical intervention [3,4]. The practice of NAET has significantly increased during the Coronavirus Disease (COVID) pandemic for early ER-positive breast cancer to rationalise resources and delay surgery in this selected group of patients. Compared to NACT, NAET has been proven to be less toxic and can be appropriate for many patients of different age groups [5].

The mechanism of action of endocrine therapy includes two main approaches. The first approach is via selective ER modulators or degraders, such as tamoxifen or fulvestrant, respectively, which target the ER itself [6]. The second approach is by inhibition of the production of oestrogen ligand; therefore, no ligand is present to activate the receptor, and this strategy is used by aromatase inhibitors. The latter blocks the aromatase enzyme and reduces the oestrogen levels in post-menopausal women. Both mechanisms abolish the activity of ER-related pathways and promote the killing of ER-dependent cancerous cells [6].

In contrast with NACT, the impacts of endocrine therapy on tumour morphology and receptor status remain poorly understood. In the adjuvant setting, the changes in the tumour morphology and immunoprofile seen in recurrent tumours could be related to tumour progression rather than to the endocrine therapy alone. The administration of neoadjuvant therapy provides an opportunity to study the biological effect encountered by such treatment, which, in the setting of NACT, is mostly characterised by alterations in the expression of hormone receptors and HER2 status [7]. 

Changes in hormone receptors and HER2 status may have a clinical impact on the management of patients following neoadjuvant treatment. In addition to being an indication of the biological response of tumours to endocrine therapy, and therefore able to be used as a potential prognosticator, the identification of a switch in receptor status may influence the planning of adjuvant systemic therapy [8,9]. Furthermore, with the recent knowledge of the response of HER2-positive breast cancer to antibody drug conjugates (ADCs), it is imperative to elucidate the effect of various therapies on HER2-low expression to inform which samples should be tested to advise management. Nevertheless, there are very little data in the literature on the effect of NAET on HER2 [10].

In this study, we aimed to evaluate the effect of NAET on the hormone receptor and HER2 status as well as the histological response of tumours utilising a multicentre study of BC patients who received NAET.

## 2. Results

### 2.1. Baseline Clinicopathologic Characteristics

Table 1 lists the pre-NAET baseline clinicopathologic features of the 391 patients in this study. The median age of the study population was 66 years (range: 29–93) and 342 patients (87.5%) were over 50 years old. The majority of patients had invasive NST carcinoma (72.9%) with a nuclear grade of 2 or 3 (80.6%). All tumours were ER-positive, of which 323 cases (82.6%) were PR-positive. HER2-0 was identified in 27.3% (n = 107), HER2-low in 67.6% (n = 264), and HER2-3+ in 5.1% (n = 20). The few patients with HER2 positivity received NAET without chemotherapy or anti-HER2 therapy, either due to patient choice or existing comorbidities. All patients received standard NAET of either tamoxifen (8.4%, n = 33) or letrozole (91.6%, n = 358). Most patients (67.5%, n = 254) underwent breast-conserving surgery.

### 2.2. Histological Response to Neoadjuvant Endocrine Therapy (NAET)

**Figure 1:**
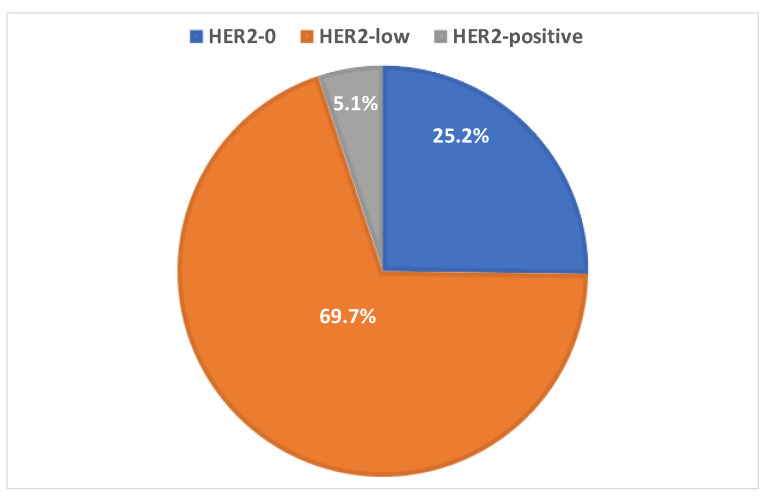
The rates of pathologic partial response (pPR) in breast cancers with baseline HER2-0, HER2-low and HER2-positive expression in this study.

Histopathologic examination of the excision specimens after NAET treatment revealed that 90.3% (n = 353) of patients achieved pPR, including 25.2% (89/353), 69.7% (246/353) and 5.1% (18/353) of the HER2-0, HER2-low and HER2-positive groups, respectively, Figure 1. The pPR rate was significantly higher in the HER2-low tumours compared to the HER2-0 and HER2-positive tumours (*p* = 0.002). Two invasive lobular carcinoma cases achieved pathological complete response.

**Figure 2:**
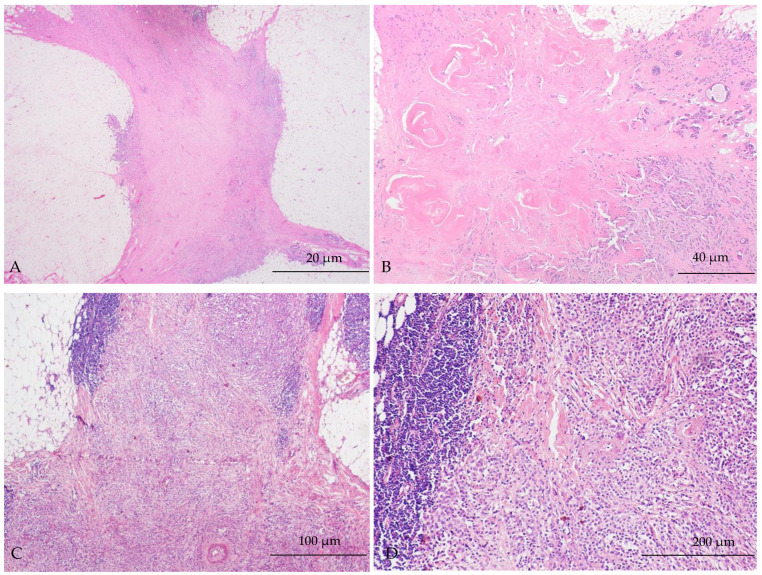
Histological changes to neoadjuvant endocrine therapy (NAET). (**A**,**B**): Residual carcinoma with prominent central scarring (magnification 2× and 4×, respectively). (**C**,**D**): Residual tumour with increased tumour infiltrating lymphocytes (TILs) (magnification 10× and 20×, respectively).

The tumours showed different histological patterns of response to NAET including central scarring, reduced cellularity and lymphocytic infiltration. The different patterns of response to NAET are shown in Figure 2.

### 2.3. Histological Tumour Type and Tumour Grade in the Matched Pre- and Post-NAET Samples

The details of the histological tumour type pre- and post-NAET are shown in Table 2. No special type (NST) carcinoma was reported in 285 (72.9%) cases on pre-NAET core biopsies compared to 268 (68.5%) cases on post-NAET excision samples. On the other hand, special type carcinoma including invasive lobular carcinoma, mucinous, papillary and micropapillary carcinoma was identified in 106 (27.1%) cases on pre-NAET core biopsies compared to 120 (30.7%) cases on post-NAET excision samples. This difference was highly statistically significant (*p* < 0.001).

**Figure 3:**
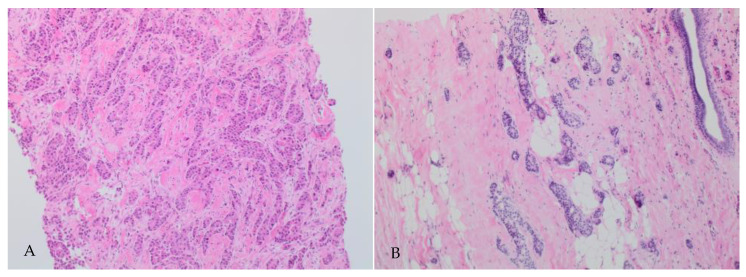
Change in grade post-NAET. (**A**) Grade 2 NST carcinoma on core biopsy. (**B**) Grade 1 residual NST carcinoma following NAET, (magnification 2× and 4×, respectively).

The histological tumour grade changes post-NAET are shown in Table 3. Tumour grading on surgical excision was not assessable for 12 cases due to the small amount of residual tumour tissue present. Grade change was noted in 30.6% (n = 116) of cancers following treatment. Downgrading (defined as a decrease in the overall grade by at least one grade) was noted in 73 out of those 116 cases (62.9%), Figure 3. This represents 19.3% out of the total gradable cases. Upgrading (defined as an increase in overall grade by at least one grade) was identified in 43 out of 116 cases (37.1%), representing 11.3% of the total number. Grade 2 and 3 tumours represented 80.5% of cases pre-NAET. This proportion dropped to 69.3% following treatment. The proportion of grade 1 tumours increased from 19.4% to 27.6% after treatment. Grade changes following NAET were statistically significant (*p* < 0.001).

### 2.4. Hormone Receptors and HER2 Status in the Paired Pre- and Post-NAET Tumours

#### 2.4.1. ER Expression

Following NAET, 4 of 387 available tumour pairs (1.03%) exhibited a change in the ER profile with a switch to ER-low positive (two cases) and ER-negative status (two cases).

#### 2.4.2. PR Expression

In the pre-NAET cohort, PR-negative, PR-low positive (1–10%) and PR-positive (>10%) tumours accounted for 13.04% (n = 51), 4.1% (n = 16) and 81.8% (n = 320), respectively. In the post-NAET cohort, PR changes were observed in 31.3% (n = 121/387) of cases. PR-negative, PR-low and PR-positive tumours accounted for 34.9% (n = 135), 7.2% (n = 28) and 57.3% (n = 224), respectively. The change in PR status following NAET was statistically significant (*p* < 0.001), with more cases losing PR expression following NAET. 

ER and PR data on surgical excision specimens were unavailable for four cases.

#### 2.4.3. HER2 Expression

A total of 302 cases were available for paired assessment of HER2 status. In the pre-NAET cohort, HER2-0, HER2-low and HER2-positive tumours accounted for 31.5% (n = 95), 62.6% (n = 189) and 5.9% (n = 18), respectively. In the post-NAET cohort, HER2 changes were noted in 38.1% (n = 115/302) of cases. HER2-0, HER2-low and HER2-positive tumours accounted for 54.9% (n = 166), 37.4% (n = 113) and 7.6% (n = 23), respectively. The changes in HER2 status pre- and post-NAET were statistically significant (*p* < 0.001), with more cases converting from a HER2-low to a HER-negative profile following NAET. 

**Figure 4:**
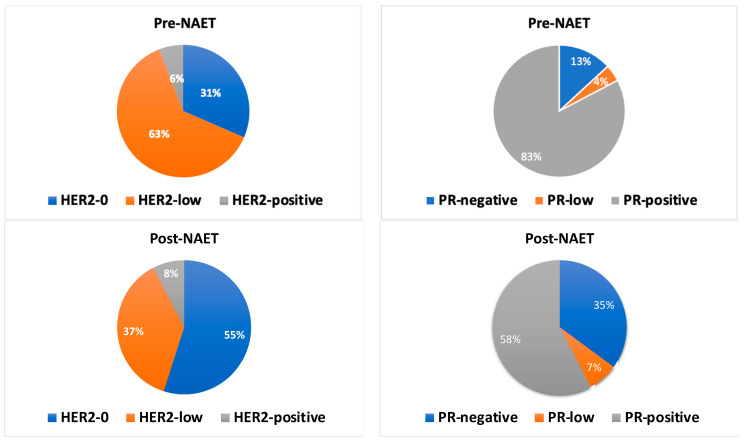
The proportion of HER2-0, HER2-low, HER2-positive, PR-negative, PR-low and PR-positive tumours in paired pre- and post-NAET tumours.

**Figure 5:**
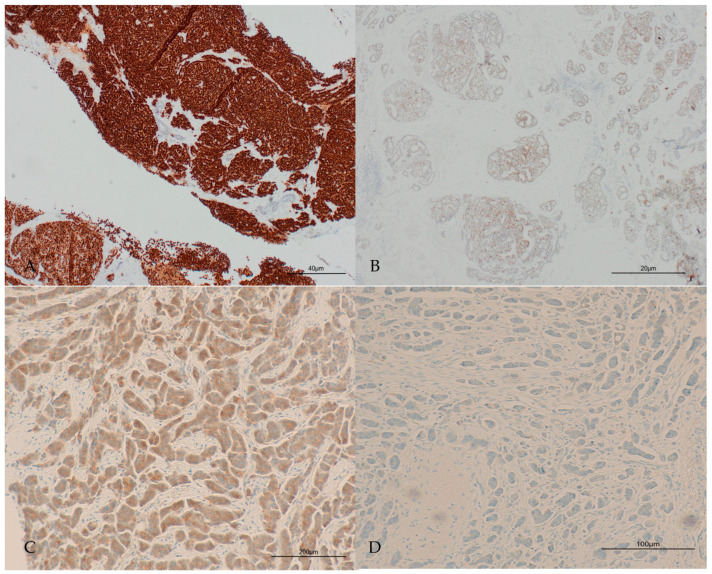
Changes in hormone receptor and HER2 status following neoadjuvant endocrine therapy (NAET). (**A**) is a core biopsy of PR-positive (Allred score 8) carcinoma that showed reduced PR expression following NAET in (**B**), (magnification 4× and 2×, respectively) (**C**) is a core biopsy of a patient with HER2-2+ carcinoma not amplified on FISH that switched to HER2-negative (score 0) following NAET in (**D**), (magnification 20× and 10×, respectively).

Table 4 shows the details of HER2 and PR status changes before and after NAET. Figure 4 shows the distribution of HER2-0, HER2-low, HER2-positive, PR-negative, PR-low and PR-positive tumours pre- and post-NAET. Figure 5 shows the changes in hormone receptor and HER2 status following NAET.

### 2.5. Ki67 Expression

Ki67 data were available for 221 post-NAET excisions. Data were not available for pre-NAET core biopsies. Tumours post-NAET showed a tendency to have a low proliferation index by Ki67, and those tumours constituted 68.33% of cases (with a cut-off value of less than 5%).

### 2.6. Relation between Biomarker Expression and Patient Outcome

The median duration of NAET was 86 days. The median patient follow-up was 246 days with an overall survival rate of 93.4%. Twenty-six patients sadly died due to complications of breast cancer. Most of the patients who died had cancers with a low HER2 profile pre-NAET (n = 20/26, 76.9%), and all except one were treated with aromatase inhibitors.

### 2.7. Relation between the Duration and Impacts of NAET

Overall, NAET had significant effects on grade, PR and HER2. However, an impact of the duration was observed for PR and HER2 expression. A longer duration of NAET correlated with a reduction in PR expression (*p* = 0.011), the conversion of HER2-low to HER2-negative status (*p* < 0.001) and a lower tumour Ki67 proliferation index (*p* = 0.006), Table 5.

## 3. Discussion

The major findings in the current study are the significant change in tumour differentiation (lower histological grade), the reduction in expression of PR and the change in HER2 status, including the conversion of HER2-low to HER2-negative status, post-NAET. Tumour differentiation showed a slight but statistically significant difference in paired pre- and post-treatment samples following NAET, with a prominence of special type ER-positive carcinomas post-NAET. This may be partly attributed to tumour heterogeneity, which may also explain some of the changes noted. It is our observation that following endocrine therapy, tumour response includes tumour cell differentiation in the form of smaller cells that start to arrange singly mimicking lobular carcinoma. Without the use of E-cadherin, these tumours can be mistakenly classified as lobular or mixed ductal and lobular. 

Without prior therapy, the current evidence shows a high concordance in tumour characteristics and receptor status, and hence, breast core biopsy is currently used for routine ER/PR/HER2 assessment [11,12,13]. Shanmugalingam et al. showed a moderate degree of agreement in grading and a strong concordance in ER and HER2 status in paired core and excision samples [12]. Ambrosini-Spaltro A and colleagues [14] reported a high concordance rate between treatment-naïve core biopsy samples and excision specimens. The small proportion of grade discordance reported in the aforementioned studies was due to underestimation of the grade due to the small sample size in core biopsy, suboptimal fixation, tissue sampled that does not include the growing edge of the tumour or intra-tumoural heterogeneity. This indicates that the grade and biomarker changes seen following NAET are likely genuine effects of therapy. 

The effect of NAET on hormone receptor expression has not been well established, and there has been great variability in the conclusions among reports [15,16]. This variability raises the clinical demand for repeat testing following neoadjuvant treatment. In the current study, four tumours (1.03%) showed a change in the ER profile with a switch to ER-low positive and ER-negative status. This finding is in line with previous reports [17,18], which showed either minimal or no change in ER status following neoadjuvant endocrine therapy.

PR losses were, however, much more common than a reduction in ER expression, consistent with previous reports [17,19], and correlated with a longer duration of NAET. The PR-negative and PR-low tumours accounted for 42% of all tumours post-NAET compared with 17.5% pre-treatment. 

It is known that different endocrine therapies work via various mechanisms to suppress the effect of oestrogen in the promotion of tumour growth [20,21]. Selective ER modulators bind to oestrogen receptors and oppose the effect of oestrogen on specific target genes [22,23]. The same effect can be achieved using selective ER degraders that degrade ERs rather than block them. In post-menopausal women, aromatase inhibitors are used to block the conversion of androgens to oestrogen with a subsequent reduction in the activity of ER [23]. During treatment, ER loss occurs in a small proportion of up to 20% of BCs, and acquisition of upregulated, over-expressed or amplified HER2 can happen instead [24,25]. By acquisition of this new property, HER2 may play a driving role in tumour progression via alternative pathways either by promoting tumour survival or reducing the level of ER, and therefore, tumours acquire endocrine resistance [26]. PR loss might also be associated with increased growth factor signalling and the upregulation of the PI3K pathway, which subsequently downregulates ER and PR expression [27]. 

In the current study, we report significant HER2 changes following NAET, with more cases showing conversion from HER2-low and HER2-positive to HER2-0 and a smaller proportion converting from HER2-0 and HER2-low to HER2-positive. A few studies have reported similar changes in the HER2 profile following endocrine treatment, with more upregulation of *HER2* than its downregulation. In one study [26], the authors reported the conversion of HER2 status from HER2-negative to HER2-positive in approximately 48.5% of their cases following a short course of NAET. They explained their finding by the presence of tumour heterogeneity, with the possibility that pre-existing *HER2* gene amplification was present and was likely not captured in the core biopsy samples [26]. However, the authors also reported downregulation of *HER3* and *HER4* genes. The downregulation of *HER3* has been shown to inhibit *HER2*-associated proliferation and tumourigenesis [28]. The HER4 nuclear domain acts as a potent ER co-expressor and promotes the proliferation of ER-positive breast tumours, whereas the *HER4* cytosolic domain has anti-proliferative and pro-apoptotic activity including tamoxifen-induced apoptosis [29]. The switch from a positive to a negative HER2 status was previously reported to be associated with worse recurrence-free survival [30]; this finding might therefore potentially indicate the need for further systemic therapy. 

HER2-low breast cancer is a new, evolving entity with prognostic implications, biological characteristics and potential for novel therapies in advanced breast cancer and metastatic disease [31]. Currently, metastatic HER2-low breast cancer is eligible for new therapeutic targets, for example, antibody drug conjugates (ADCs) such as Trastuzumab and Deruxetecan [31]. We have shown, for the first time, a significant proportion of tumours switching from HER2-low status to HER2-0 following NAET. 

It is worth noting that even with the hormone receptor status changes following NAET from positive to negative status, patients may still benefit from adjuvant endocrine therapies [32]. Therefore, we recommend that endocrine therapy should still be considered for patients showing only one positive hormone receptor status either pre- or post-NAET. 

Ki67 has been used as a marker for assessing tumour proliferation [33]. A few studies have shown that Ki67 proliferation status changes following neoadjuvant therapy, with a reduction in the proliferation index [34]. This finding has been shown to correlate with the long-term outcome after NAET. The Ki67 working group suggested that in the neoadjuvant setting, the Ki67 status could be used as an indicator for the early determination of endocrine therapy efficacy (pre-NAET) and could have value for late determination (post-NAET) [35]. In the current study, we have shown that 68% of tumours had a low proliferation index based on Ki67 following a course of neoadjuvant endocrine therapy and that the presence of a low proliferation index was associated with a longer duration of therapy. 

In addition to the biomarker expression, morphological changes in response to NAET were observed in the current study. These included central scarring, reduced cellularity and the presence of tumour-infiltrating lymphocytes. This is in line with the previously reported, though scarcely described, morphological changes post-neoadjuvant endocrine treatment. In their study, Thomas et al. [36] were the first to describe central scarring as a feature of NAET response compared to NACT. The presence of central scarring was a feature of the response to aromatase inhibitors, and this was significantly associated with a reduction in tumour size and the clinical response. A similar finding was further supported by Badr et al., who reported central scarring in approximately half of their cohort [37].

## 4. Materials and Methods

### 4.1. Patient Selection

This was a retrospective study that included female patients (n = 391) who received neoadjuvant endocrine therapy (NAET) for primary operable ER-positive invasive breast cancer between October 2012 and October 2020, with subsequent therapeutic surgery. Cases with missing information regarding the hormone receptor status of the post-treatment excision sample, patients treated with neoadjuvant chemotherapy (NACT) or patients who did not have subsequent surgery were excluded.

### 4.2. Data Collection

This was a multicentre study of patients treated at 7 UK contributing institutions. The baseline (pre-NAET) clinicopathologic characteristics of 391 patients were collected, including age at initial diagnosis; tumour histological type; tumour grade; and ER, PR and HER2 status. The latter was subclassified into the HER2-0, HER2-low and HER2-positive categories. The NAET regimen, tumour histological response to NAET, post-NAET HER2 and hormone receptor status for cases with residual disease and clinical follow-up information were also recorded. 

The response to treatment was classified into pathological complete response (pCR), i.e., no residual invasive carcinoma in the breast and in the axillary lymph nodes; minimal residual disease (≤10% residual invasive carcinoma); pathological partial response (pPR), i.e., >10% residual invasive carcinoma; and no response (no histological evidence of tumour response), as previously described [37].

Cases with no repeat testing for ER, PR or HER2 were immunohistochemically stained, where possible, and manual scoring was performed by a breast pathology specialist to obtain the missing information.

### 4.3. Hormone Receptors and HER2 Testing and Re-Evaluation

All paired pre- and post-NAET ER, PR and HER2 IHC-stained slides were evaluated. The hormone receptor status was considered positive if immunohistochemical staining for ER and PR was present in ≥1% of invasive tumour cell nuclei. ER and PR were considered low if positivity was achieved in 1–9% of tumour cell nuclei. The HER2 evaluation was performed by adhering to both the UK guidelines as well as the 2023 ASCO/CAP HER2 testing guidelines [38,39,40]. HER2 score 0 was defined as no staining observed or incomplete membranous staining that was faint or barely perceptible in ≤10% of invasive tumour cells. HER2 score 1+ was considered when there was incomplete faint or barely perceptible membrane staining in >10% of invasive tumour cells. HER2 score 2+ was defined as weak to moderate complete membrane staining detected in >10% of invasive tumour cells. HER2 score 3+ was defined as complete and strong complete membrane staining in >10% of invasive tumour cells. HER2 positivity was defined as an immunohistochemistry score of 3+ or a 2+ fluorescence in situ hybridisation (FISH)-amplified score as per the UK guidelines [41]. A HER2 immunohistochemistry score of 1+ or a 2+ score non-amplified on FISH was regarded as HER2-low breast cancer. A HER2-0 score was considered negative [42].

### 4.4. Statistical Analysis

Statistical analysis was performed using IBM SPSS package v.29. Analyses of the categorical data, including the tumour type, grade, hormone receptor status and HER2 expression, were performed using the Z-test or Chi-square test (X^2^). The non-parametric Mann–Whitney and Kruskal–Wallis tests were used to assess the relationship between the duration of NAET as a continuous variable and the different clinicopathological parameters.

Overall survival (OS) was defined as the duration in months between the date of diagnosis and the date of last follow-up or death. A *p*-value of ≤0.05 was considered statistically significant

## 5. Conclusions

In conclusion, we show that neoadjuvant endocrine therapy resulted in morphological and biomarker changes, and these changes were more pronounced as the duration of therapy increased. There was a tendency for the downgrading and conversion of HER2 and PR status following treatment. The changes in hormone receptors and HER2 might be of prognostic value and predictive importance and may provide further options for therapeutic targets. The increase in the TILs is an intriguing feature that requires further analysis and may reflect a complex interaction between the epithelial cells and the tumour microenvironment. We hereby recommend a thorough histological assessment of the post-excision residual invasive tumour following NAET as well as repeat testing of hormone receptors and HER2.

### Study Strengths and Weakness

This is a UK multicentre study that analysed the effect of NAET on a well-characterised large cohort of ER-positive breast cancers with particular emphasis on receptor expression. We have shown significant changes in biomarker expression with a particular focus on the low-PR and low-HER2 group evolution. Despite the strengths of the current study, there are a few limitations. These include the retrospective multicentre nature of the study with the inevitable unavailability of some data. The lack of Ki67 data on the pre-treatment diagnostic samples limited the comparison of the proliferation index before and after NAET.

## Figures and Tables

**Table 1:** Baseline pre-neoadjuvant endocrine therapy (pre-NAET) clinicopathological features of 391 patients included in this study.

Demographics	Number	%
Age (years, median, range)	66 (29–93)	
<50 Years	49	12.5
≥50 Years	342	87.5
Ethnic origin		
White British	199	50.9
Scottish	45	11.5
Asian	15	3.8
Any other mixed	16	4.1
Unknown	116	29.7
Histologic tumour type		
Ductal NST	285	72.9
Lobular	71	18.2
Mixed	13	3.3
Other	22	5.6
Nuclear grade		
I	76	19.4
II	275	70.3
III	40	10.3
Tubule formation		
1	18	4.6
2	106	27.1
3	267	68.3
Pleomorphism		
1	1	0.3
2	255	65.2
3	135	34.5
Mitosis		
1	332	84.9
2	43	11.0
3	16	4.1
Biomarker status		
ER-positive/HER2-0	107	27.3
ER-positive/HER2-low	264	67.6
ER-positive/HER2-positive	20	5.1
PR status		
Negative	51	13.1
Low	17	4.3
Positive	323	82.6
NAET		
Tamoxifen	33	8.4
Letrozole	358	91.6
Type of surgery		
Breast-conserving surgery	254	67.5
Mastectomy	127	32.5
Pathologic response		
pCR	2	0.5
pPR	353	90.3
Minimal residual disease	24	6.1
No response	12	3.1

Abbreviations: ER, Oestrogen receptor; PR, Progesterone receptor; HER2, Human epidermal growth factor receptor 2; NAET, Neoadjuvant endocrine therapy; pCR, Pathologic complete response; pPR: Pathologic partial response.

**Table 2:** Details of tumour types before and after NAET.

Pre-NAET Tumour Type	Post-NAET Tumour Type					Total	X^2^ *p* Value	Cohen’s Kappa Value
	NST	ILC	Mixed Ductal and Lobular	Other *	No Residual Tumour			
NST	252 (64.5)	6 (1.5)	9 (2.3)	17 (4.4)	1 (0.3)	285	462.06 **<0.001**	0.668 **<0.001**
ILC	5 (1.3)	60 (15.3)	4 (1.02)	0 (0)	2 (0.5)	71		
Mixed ductal and lobular	4 (1.02)	1 (0.3)	7 (1.8)	1 (0.3)	0 (0)	13		
Other *	7 (1.8)	2 (0.5)	1 (0.3)	12 (3.1)	0 (0)	22		
Total	268	69	21	30	3	391		

Abbreviations: NAET, Neoadjuvant endocrine therapy; NST, No special type; ILC, Invasive lobular carcinoma. * This includes tubular, mucinous, papillary and micropapillary carcinoma. Bold *p*-values are significant.

**Table 3:** Details of tumour grade distribution in pre-treatment biopsies and post-treatment residual invasive carcinoma.

Pre-NAET Grade	Post-NAET Grade						
	I	II	III	Unknown	Total	X^2^ (*p* Value)	Cohen’s Kappa Value
I	56 (14.3)	17 (4.3)	1 (0.3)	2 (0.5)	76	131.924 **<0.001**	0.400 **<0.001**
II	49 (12.5)	192 (49.1)	25 (6.4)	9 (2.3)	275		
III	3 (0.8)	21 (5.8)	15 (3.8)	1 (0.3)	40		
Total	108	230	41	12	391		

Abbreviations: NAET, neoadjuvant endocrine therapy. Bold *p*-values are significant.

**Table 4:** Details of HER2 and PR status changes before and after NAET.

**Pre-NAET HER2 Status**	(**A**) **Post-NAET HER2 Status**						**X^2^** **(*p* Value)**	**Cohen’s ** **Kappa Value**
	**HER2-0**	**HER2-1+**	**HER2-2+**	**HER2-3+**	**Unknown**	**Total**		
HER2-0	80 (20.5)	5 (1.3)	9 (2.3)	1 (0.3)	12 (3.1)	95	47.382 **<0.001**	0.375 **<0.001**
HER2-1+	46 (11.8)	25 (6.4)	8 (2.0)	3 (0.8)	61 (15.6)	143		
HER2-2+	39 (10.0)	14 (3.6)	59 (15.1)	3 (0.8)	16 (4.1)	131		
HER2-3+	1 (0.3)	1 (0.3)	0 (0.0)	8 (2.0)	0 (0.0)	10		
Total	166	45	76	15	89	391		
**Pre-NAET HER2 Final Status**	(**B**) **Post-NAET HER2 Final Status**						**X^2^** **(*p* Value)**	**Cohen’s** **Kappa Value**
	**HER2-0**		**HER2-Low**	**HER2-Positive**	**Total**			
HER2-0	80 (20.5)		14 (3.6))	1 (0.3)	95		47.382 **<0.001**	0.364 **<0.001**
HER2-Low	82 (21.0)		97 (24.8)	10 (2.6)	189			
HER2-Positive	4 (1.0)		2 (0.5)	12 (3.1)	18			
Total	166		113	23	302			
**Pre-NAET PR Status**	(**C**) **Post-NAET PR Status**						**X^2^** **(*p* Value)**	**Cohen’s** **Kappa Value**
	**Negative**		**Low**	**Positive**	**Total**			
PR-Negative	43 (11.0)		3 (0.8)	5 (1.3)	51		79.916 **<0.001**	0.316 **<0.001**
PR-Low	10 (2.6)		3 (0.8)	3 (0.8)	16			
PR-Positive	82 (21.0)		22 (5.6)	216 (55.2)	320			
Total	135		28	224	387			

HER2, Human epidermal growth factor receptor 2; NAET, Neoadjuvant endocrine treatment; X^2^: Chi square. Bold *p*-values are significant.

**Table 5:** Effect of the duration of neoadjuvant endocrine therapy and the clinicopathological parameters.

Parameter	Number (%)	Mean Rank	*p* Value
Tumour type change			**<0.001**
-No	331 (85.3)	186.02	
-Yes	57 (14.7)	243.73	
Grade change			**0.029**
-No	263 (67.8)	180.15	
-Upgrade	43 (11.1)	217.37	
-Downgrade	43 (11.1)	209.38	
ER status change			0.556
-No	383 (98.7)	193.66	
-Yes	4 (1.1)	226.75	
ER status			0.793
-Negative	2 (0.5)	246.00	
-Low	2 (0.5)	207.50	
-Positive	383 (98.7)	193.66	
PR status change			0.101
-No	266 (68.6)	187.70	
-Yes	121 (31.2)	207.84	
PR status			**0.011**
-Negative	135 (34.8)	217.36	
-Low	28 (7.2)	176.86	
-Positive	224 (57.7)	182.07	
HER2 status change			**0.014**
-No	187 (48.2)	161.23	
-Yes	115 (29.6)	135.68	
HER2 status			**<0.001**
-Negative	166 (42.8)	128.79	
-Low	113 (29.1)	184.21	
-Positive	23 (5.9)	154.72	
Ki67			**0.006**
-Low Proliferation	151 (38.9)	119.05	
-High Proliferation	70 (18.1)	93.64	

PR: Progesterone receptor; HER2: Human epidermal growth factor receptor 2; ET: Endocrine therapy. Bold *p*-values are significant.

## Data Availability

Data are contained within the article.
